# Current situation and trend of non-coding RNA in rheumatoid arthritis: a review and bibliometric analysis

**DOI:** 10.3389/fimmu.2023.1301545

**Published:** 2024-01-16

**Authors:** Zehong Wei, Huaiyu Li, Senhao Lv, Junping Yang

**Affiliations:** ^1^ Graduate School, Jiangxi University of Chinese Medicine, Nanchang, Jiangxi, China; ^2^ Clinical Laboratory, Affiliated Hospital of Jiangxi University of Chinese Medicine, Nanchang, Jiangxi, China

**Keywords:** bibliometrics, visualization, rheumatoid arthritis, non-coding RNA, hotspots

## Abstract

**Background:**

Rheumatoid arthritis (RA) is a chronic, systemic autoimmune disease that affects multiple joints and has adverse effects on various organs throughout the body, often leading to a poor prognosis. Recent studies have shown significant progress in the research of non-coding RNAs (ncRNAs) in RA. Therefore, this study aims to comprehensively assess the current status and research trends of ncRNAs in RA through a bibliometric analysis.

**Methods:**

This study retrieved articles relevant to ncRNAs and RA from the Science Citation Index Expanded Database of the Web of Science Core Collection between January 1st, 2003, and July 31st, 2023. The relevant articles were screened based on the inclusion criteria. VOSviewer and CiteSpace are utilized for bibliometric and visual analysis.

**Results:**

A total of 1697 publications were included in this study, and there was a noticeable increase in annual publications from January 1st, 2003, to July 31st, 2023. China, the United States, and the United Kingdom were the most productive countries in this field, contributing to 43.81%, 13.09%, and 3.87% of the publications. Anhui Medical University and Lu Qianjin were identified as the most influential institution and author. Frontiers In Immunology stood out as the most prolific journal, while Arthritis & Rheumatology was the most co-cited journal. Additionally, the research related to “circular RNA”, “oxidative stress”, “proliferation”, and “migration” have emerged as new hotspots in the field.

**Conclusion:**

In this study, we have summarized the publication characteristics related to ncRNA and RA and identified the most productive countries, institutions, authors, journals, hot topics, and trends.

## 1 Introduction

Rheumatoid arthritis (RA) is a chronic autoimmune disease characterized by symmetric polyarthritis. RA exhibits synovial inflammation which is the fundamental pathological alteration in the early stage of disease, then synovium proliferates and pannus forms that damages joints and impairs joint function, ultimately results in disability (1). RA is a major global public health challenge, according to the global epidemiological study on RA in 2017, the age-standardized global prevalence and annual incidence rates of RA were 0.246% and 0.015%, which show an increasing trend (2, 3). The etiology of RA has not yet been fully clarified, but it generally involves a complex interplay among genetic susceptibility, environmental exposures, microbial infections, and immune dysregulation (4, 5). American college of rheumatology guideline for the treatment of rheumatoid arthritis indicates that the treatment of RA includes nonsteroidal anti-inflammatory drugs (NSAIDs), glucocorticoids (GC), conventional synthetic disease-modifying antirheumatic drugs (csDMARDs), biologic DMARDs (bDMARDs) and targeted synthetic DMARDs (tsDMARDs), as well as joint replacement surgery (6, 7).However, RA cannot be cured entirely, and it is essential to elucidate the pathogenesis of RA to update therapeutic strategies and develop novel medicine.

Non-coding RNAs (ncRNAs) which are not translated to protein have a crucial role in regulating gene expression, mainly include microRNA (miRNA), long non-coding RNA (lncRNA), and circular RNA (circRNA) (8). In recent years, an increasing number of genetic studies related to the pathogenesis of RA (including gene-arrays and new generation sequencing studies) revealed gene expression signatures and networks in synovial tissue and peripheral blood mononuclear cells (PBMCs) (9–11). Furthermore, research on post-transcriptional mechanisms of gene regulation has identified ncRNAs associated with disease activity, inflammation, pannus formation and bone destruction (12). These ncRNAs play a significant role in the diagnosis and treatment of RA as biomarkers, mediators of pathogenesis and potential therapeutic targets (12, 13).

Bibliometrics is an important method of quantitatively and qualitatively analyzing publications within a specific field via mathematical and statistical approaches (14). It aims to uncover the research hotspots and trends by acquiring, processing, and analyzing the data of countries, institutions, author, journals, and keywords (15). Despite the intrinsic mechanisms of ncRNAs in the pathogenesis of RA have been continuously unveiled over the past two decades, there have no bibliometric analysis has been published focusing on the field. In this study, we aimed to use bibliometric method and mapping knowledge domain through softwares (such as VOSviewer (16) and CiteSpace (17)), to visually analyze the publications related to ncRNAs in RA, and identify the global research status, trends, and hotspots in the field.

## 2 Materials and methods

### 2.1 Data collection

**Figure 1:**
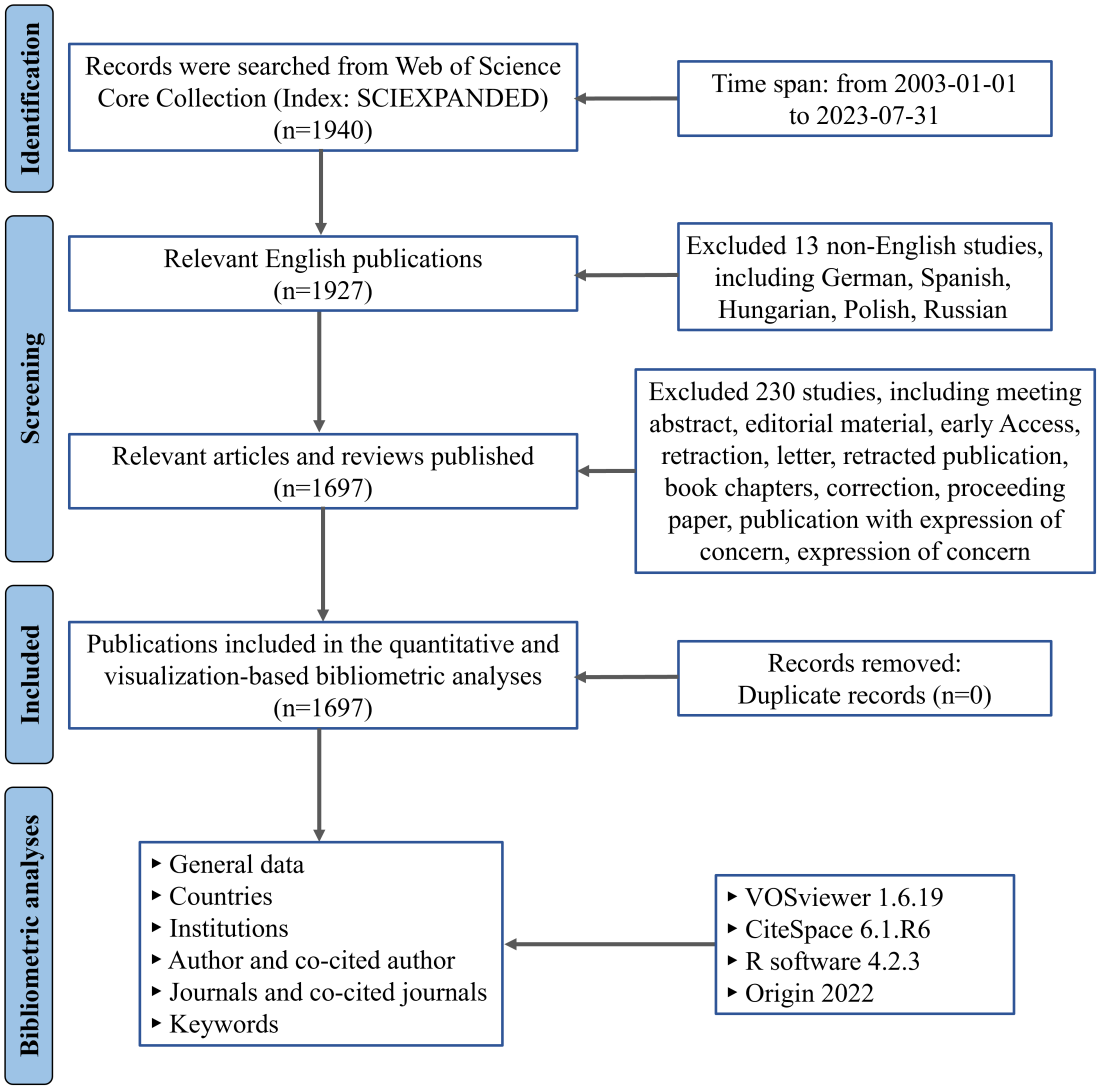
Flowchart for the research’s search procedure.

In this study, we retrieved and downloaded the data from the Science Citation Index Expanded (SCI-Expanded) Database of the Web of Science Core Collection (WoSCC) on August 20, 2023. The procedure for data retrieval and collection is illustrated in **Figure 1**. The search strategy was as follows: TS=((“rheumatoid arthritis”) AND (“non-coding RNA” OR “noncoding RNA” OR miR-* OR miRNA$ OR microRNA$ OR circ_* OR circRNA$ OR Circular RNA$ OR lnc-* OR lncRNA$ OR LincRNA$ OR “long noncoding RNA” OR “long non-coding RNA” OR “long intergenic non-coding RNA” OR “long ncRNA”)), and the timespan of the index date was set from January 1st, 2003, to July 31st, 2023. Afterwards, the document types were limited to “articles” and “reviews”, and the language type of documents was restricted to English. The full record and cited references of all retrieved documents were saved as “plain text” files which were used for further analysis. Data including countries, institutions, authors and co-cited authors, journals and co-cited journals, as well as Hirsch’s hybrid index (H-index) were extracted from the included publications.

### 2.2 Visualized analysis

The Web of Science (WOS) database is widely recognized as the primary source of citation information in bibliometric research, providing general statistical data including publication year, citation count, H-index, etc (18, 19). VOSviewer (version 1.6.19), CiteSpace (version 6.1. R6), R (version 4.2.3), and Origin 2022 were used to analyze data and generate visualization map.

R was used to generate geographical distribution map based on the total publications of different countries between January 1st, 2003, and July 31st, 2023. Origin was used to generate the trend chart of global publications and citations for each year, and the international collaborations’ chord diagram of countries. VOSviewer was used to generate visualization map and analyze the co-occurrence of productive countries, institutions, authors and co-authors, journals and co-cited journals. CiteSpace was used to construct the superposition of journal double maps and the keyword network visualization map from the time zone perspective to display the evolution of research hotspots over time.

In the visualization map, node size is correlated with the type of data being analyzed, typically determined by factors such as the counts of publications, the frequency of occurrence, or the number of citations. Nodes representing correlated data have connecting lines and share the same color. The lines generally represent the cooperation between countries, the association between keywords, or other relevant connections, and the thickness of line indicates the strength of cooperation, which was assessed by total link strength (TLS).

### 2.3 Research ethics

This study does not require ethical approval because it does not involve human or animal experiments, and the data was obtained from publicly accessible databases.

## 3 Results

### 3.1 Global trend in publications and citations

**Figure 2:**
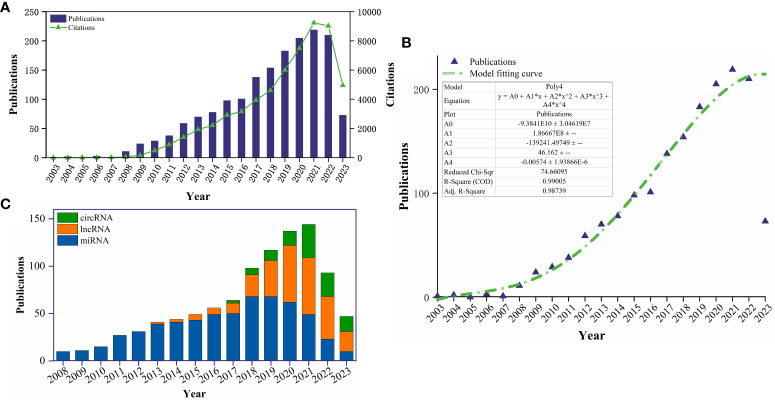
**(A)** The global annual number of publications and citation on ncRNA-related RA research from 2003-01-01 to 2023-07-31; **(B)** Model fitting curves of global trends in publications; **(C)** The annual publication trends related to the main ncRNAs (miRNA, lncRNA, and circRNA) in RA.

After excluding 243 publications that did not meet the inclusion criteria, a total of 1697 publications between January 1st, 2003, and July 31st, 2023, were obtained from WoSCC database, including 1272 articles and 412 reviews. **Figure 2A** shows the global trend in publications and total citations for ncRNA-related RA research from January 1st, 2003, to July 31st, 2023. The annual publication count has shown a growing trend over the past two decades, with particularly notable growth in the last five years, during which more than half of all publications were released. All publications have been cited 58522 times as of the search date, with 34.49 average citations per document. **Figure 2B** illustrates a polynomial fitting curve (R^2 =^ 0.99005) depicting the annual growth trend of publications. As of date (July 31, 2023), there have been over 73 articles published in 2023, with the publication count steadily increasing. Overall, these findings indicate that research on RA-related ncRNA remains highly innovative. **Figure 2C** shows the annual publication trends related to the main types of ncRNAs (miRNA, lncRNA, and circRNA) in RA. It can be observed that the focus of ncRNA research in the RA field is gradually shifting from miRNA to lncRNA and circRNA. Although there has been an overall decline in the number of ncRNA publications in the field of RA in recent years due to the impact of the COVID-19 pandemic, it is expected that future research on ncRNAs in RA will primarily focus on lncRNA and circRNA and continue to increase.

### 3.2 Analysis of countries of the publications

**Figure 3:**
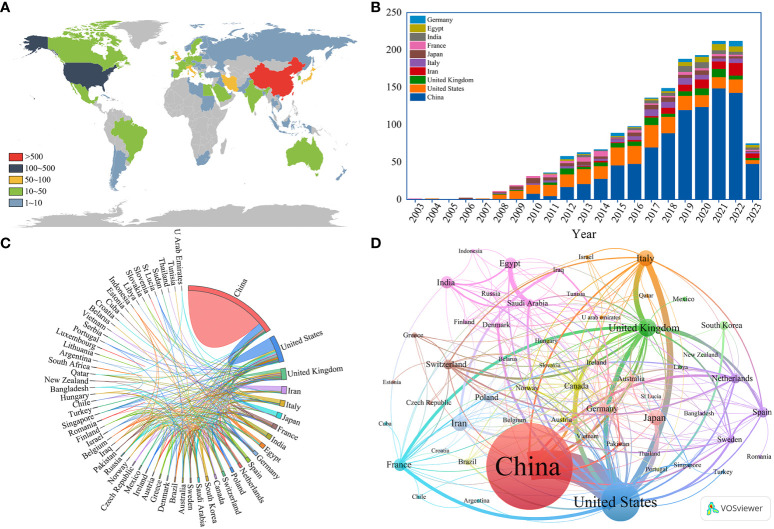
**(A)** Geographical distribution map based on the total publications of different countries; **(B)** The trend in annual publication counts of the top 10 most productive countries throughout the period 2003-01-01~2023-07-31; **(C)** The international collaborations’ visualization map of countries; **(D)** The co-authorship network visualization map of countries generated by VOSviewer.

A total of 61 different countries have published publications relating to this topic. According to the geographical distribution map based on the total publications of different countries (**Figure 3A**), documents related to ncRNA-related RA research were primarily published in China and the United States. **Table 1** lists the top ten most productive countries regarding ncRNA and RA research. China had most publications (917, 43.81%), followed by the United States (203, 13.09%), and the United Kingdom (81, 3.87%). **Figure 3B** shows the trend in annual publication counts of the top 10 most productive countries throughout the period from January 1st, 2003, to July 31st, 2023, and China has the highest annual published growth rate. Moreover, China has most total citations (21811) and the highest H-index (69), indicating China’s positive influence and importance in the field of ncRNA research for RA. **Figures 3C, D** depict the international cooperation among different countries, the United States had the strongest international cooperation network (TLS=203) and cooperated most closely with China (TLS=113).

**Table 1:** The top 10 most productive countries regarding ncRNA and RA research from 2003-01-01 to 2023-07-31.

Rank	Country	Counts	Percentage	TLS	Total citations	Average citation per paper	H-index
1	China	917	43.81%	113	21811	23.7852	69
2	United States	274	13.09%	203	17092	62.3796	68
3	United Kingdom	81	3.87%	98	3696	45.6296	34
4	Iran	70	3.34%	27	1506	21.5143	23
5	Italy	67	3.20%	55	3113	46.4627	26
6	Japan	65	3.11%	25	5143	79.1231	33
7	France	47	2.25%	40	2246	47.7872	25
8	India	44	2.10%	23	1042	23.6818	17
9	Egypt	40	1.91%	25	600	15	14
10	Germany	37	1.77%	45	1151	31.1081	18

### 3.3 Analysis of institutions of the publications

**Figure 4:**
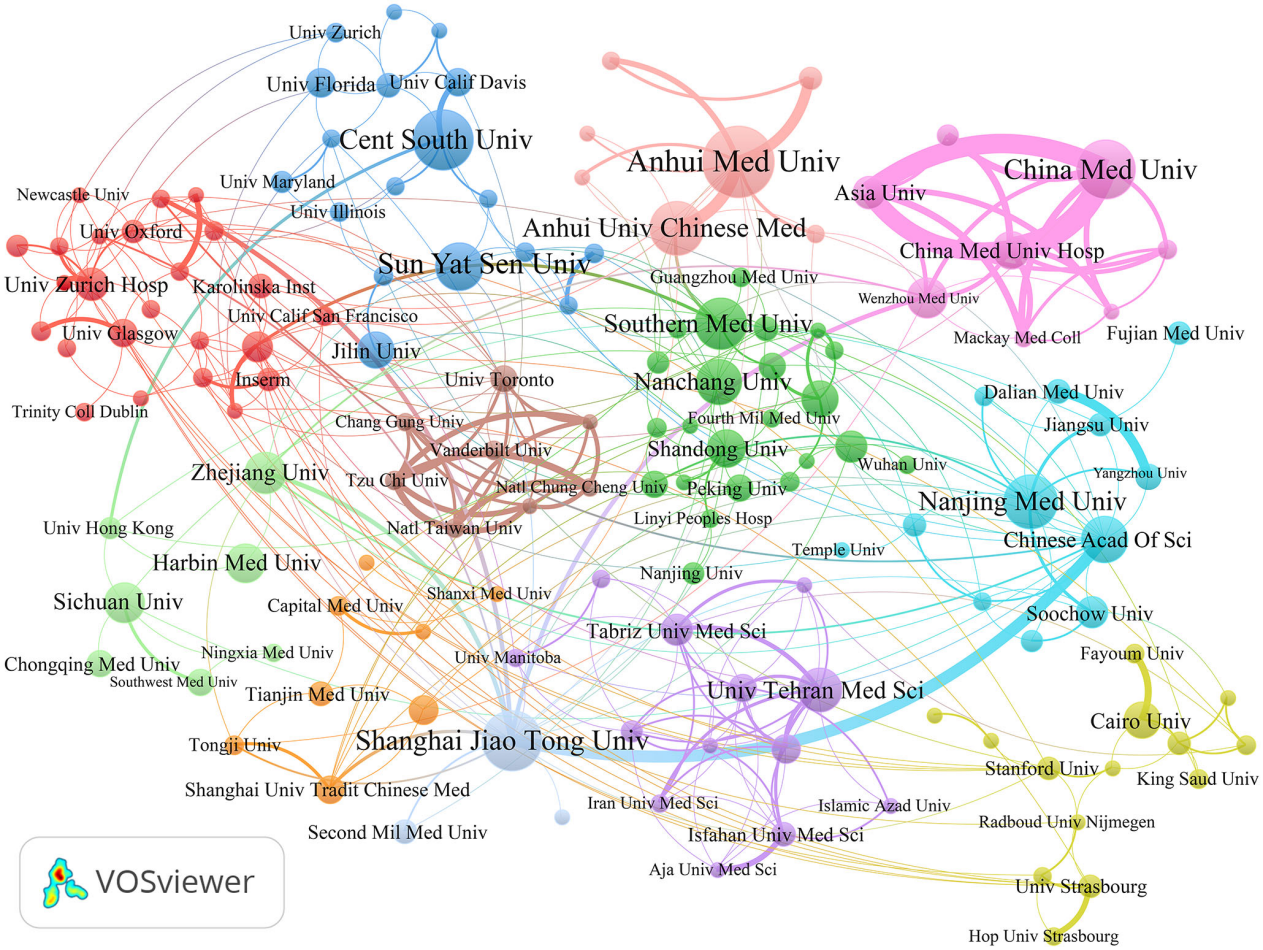
The co-authorship network visualization map of institutions generated by VOSviewer.

A total of 1827 institutions have contributed to the publications on ncRNA-related RA research. **Table 2** shows the top 10 most productive institutions, while **Figure 4** illustrates the co-authorship network visualization map of institutions. China has conducted extensive research on ncRNA in RA. The top ten most productive institutions are from China, contributing 345 articles, accounting for 20.57% of the total publications. Most articles were produced by Anhui Medical University, Central South University, Shanghai Jiao Tong University, and China Medical University. Central South University had the highest H-index (23), followed by Anhui Medical University (22) and Shanghai Jiao Tong University (22). Additionally, Shanghai Jiao Tong University had the highest number of total citations, while the Chinese Academy of Sciences had the highest average number of citations per paper. Institutions with at least five publications were presented in the co-authorship network visualization map (**Figure 4**), which contains 161 nodes and 1478 linkages. China Medical University (TLS = 91), Shanghai Jiao Tong University (TLS = 83), and Anhui Medical University (TLS = 65) are the top three institutions with the highest TLS.

**Table 2:** The top 10 most productive institutions regarding ncRNA and RA research from 2003-01-01 to 2023-07-31.

Rank	Institutions	Counts	Country	TLS	Total citations	Average citation per paper	H-index
1	Anhui Medical University	52	China	65	1480	28.4615	22
2	Central South University	39	China	25	1935	49.6154	23
3	Shanghai Jiao Tong University	39	China	83	2376	60.9231	22
4	China Medical University	38	China	91	1008	26.5263	20
5	Nanjing Medical University	34	China	44	777	22.8529	15
6	Anhui University Of Chinese Medicine	33	China	46	385	11.6667	12
7	Southern Medical University	31	China	40	724	23.3548	17
8	Sun Yat Sen University	28	China	23	641	22.8929	12
9	Xi An Jiaotong University	28	China	19	521	18.6071	13
10	Chinese Academy Of Sciences	27	China	54	2086	77.2593	17

### 3.4 Analysis of authors and co-cited authors of the publications

**Figure 5:**
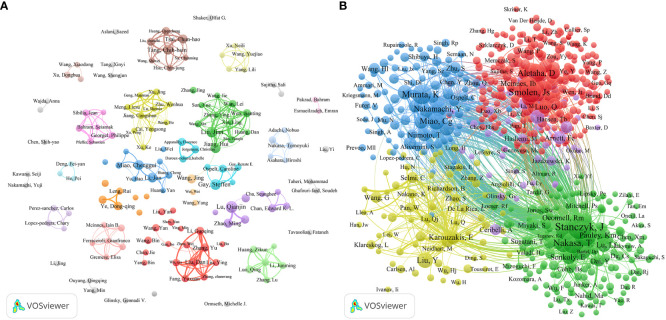
The co-authorship network visualization map of authors **(A)** and co-cited authors **(B)** generated by VOSviewer.

This study investigates 8589 authors and 40806 co-cited authors (**Figure 5**). **Table 3A** shows the top ten most productive authors regarding ncRNA and RA research. Lu, Qianjin (from Central South University) had most publications (21), followed by Gay, Steffen (from University Zurich Hospital) with 19 documents, and Liu, Jian (from Anhui University of Chinese Medicine) with 16 documents. In addition, Lu, Qianjin also had the highest H-index (19), while Gay, Steffen had the most total citations and the highest average number of citations per paper. Their research has a significant impact on the field of ncRNA in RA. Authors who are simultaneously referenced by two (or more) authors in one or even more publications are known as co-cited authors. **Table 3B** shows the top ten co-cited authors. Stanczyk, J (441 citation), Pauley, Km (386 citation), and Nakasa, T (314 citation) are the top three co-cited authors. They had made an indispensable contribution to the knowledge base of ncRNA-related RA research.

**Table 3a:** The top 10 most productive authors regarding ncRNA and RA research from 2003-01-01 to 2023-07-31.

Rank	Author	Counts	Total citations	Average citation per paper	Institutions	H-index
1	Lu, Qianjin	21	1132	53.9	Central South University	19
2	Gay, Steffen	19	2312	123.68	University Zurich Hospital	17
3	Liu, Jian	16	104	6.5	Anhui University of Chinese Medicine	6
4	Miao, Chenggui	15	324	21.6	Anhui University of Chinese Medicine	10
5	Tang, Chih-Hsin	14	472	33.71	China Medical University	12
6	Tsai, Chun-Hao	13	471	36.23	China Medical University	12
7	Ye, Dongqiang	11	542	49.27	Anhui Medical University	10
8	Wu, Haijing	11	475	43.18	Central South University	11
9	Selmi, Carlo	11	267	24.27	Humanitas University	8
10	Pan, Hai-Feng	10	520	52	Anhui Medical University	9

**Table 3b:** The top 10 co-cited authors regarding ncRNA and RA research from 2003-01-01 to 2023-07-31.

Rank	Co-cite author	Citations	TLS
1	Stanczyk, J	441	32924
2	Pauley, Km	386	25737
3	Nakasa, T	314	21672
4	Oconnell, Rm	304	26556
5	Smolen, Js	303	21683
6	Bartel, Dp	276	20511
7	Murata, K	265	20653
8	Mcinnes, Ib	248	16737
9	Aletaha, D	227	12372
10	Taganov, Kd	221	16238

### 3.5 Analysis of journals and co-cited journals of the publications

The top ten most productive journals contributed 327 articles, accounting for 19.27% of the total publications. Frontiers In Immunology (n=73), International Journal Of Molecular Sciences (n=41), Plos One (n=32) are the top three academic journals that publish articles on ncRNA-related RA research out of 504 academic journals. Frontiers In Immunology also had the highest H-index (26), followed by Arthritis Research & Therapy (24) and Plos One (21). Additionally, Arthritis Research & Therapy had the most total citations and the highest average number of citations per paper.

The journal impact factor (IF) is also one of the indicators used to assess the influence of academic journals, including measuring the journal’s reputation, evaluating research quality, and assessing the impact of research outcomes (20). Journal Of Autoimmunity had the highest impact factor (IF 2023 = 12.8), followed by Frontiers In Immunology (IF 2023 = 7.3) and International Journal Of Molecular Sciences (IF 2023 = 5.6). **Figure 2B** shows the co-occurrence network visualization map of co-cited journals. The top three co-cited journals are Arthritis & Rheumatology (4696 citations), Journal Of Immunology (2457 citations), Arthritis Research & Therapy (2447 citations).

**Figure 6:**
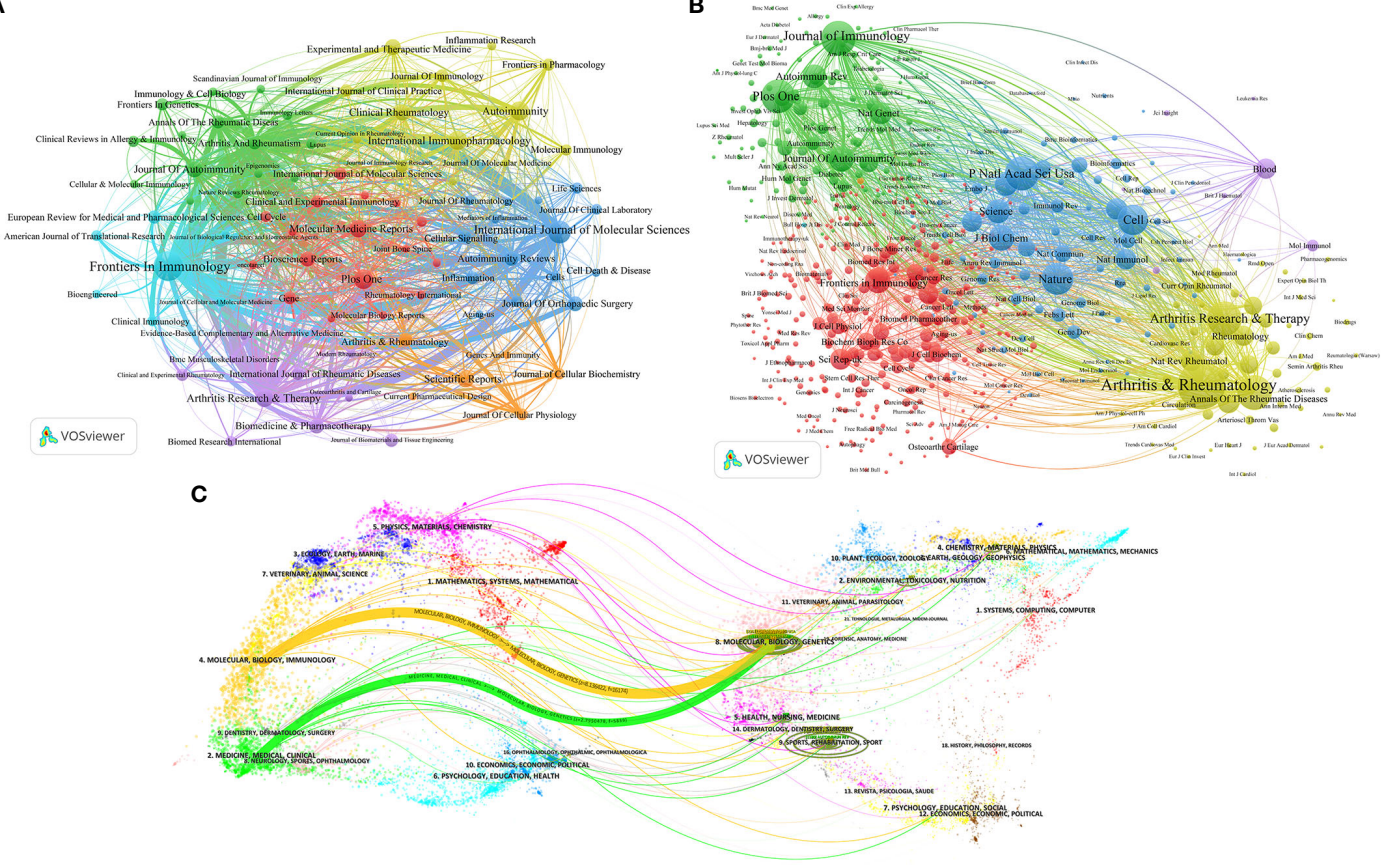
The co-occurrence network visualization map of journals **(A)** and co-cited journals **(B)** generated by VOSviewer; **(C)** The superposition of journal double maps.

The superposition of journal double maps (**Figure 6C**) shows the distribution of journals’ topics. Various research fields covered by all the journals are displayed on the labels of the map. The citing journals are displayed on the left side of the map, while the cited journals are shown on the right side. The connecting lines represent that research in certain topics of the cited journals is often cited by research in certain topics of the citing journals, and the width of the lines is closely related to the frequency of citations. There were two main connecting lines on **Figure 6C**, indicating that research published in the Molecular/Biological/Immunology journals and the Medicine/Medical/Clinical journals generally referenced studies published in the Molecular/Biology/Genetics journals.

### 3.6 Analysis of keywords of the publications

**Figure 7:**
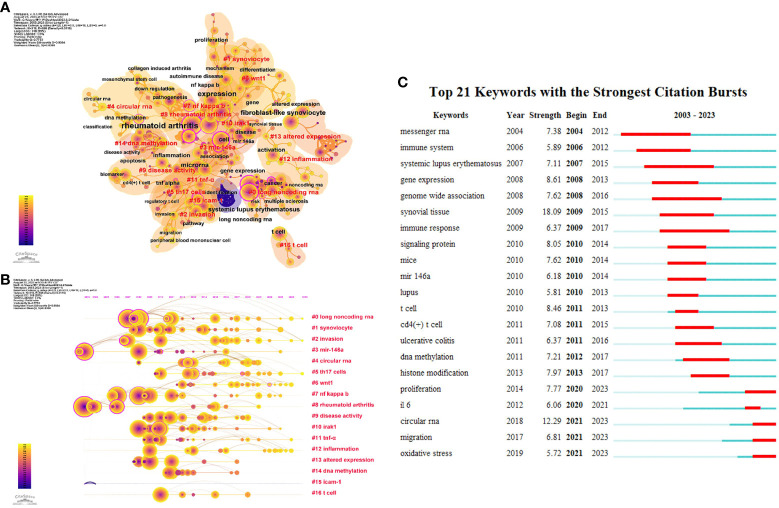
The co-occurrence network visualization map of keywords in Cluster view **(A)** and timeline view **(B)** generated by CiteSpace; **(C)** The top 21 keywords with the strongest citation bursts.

Keyword co-occurrence analysis is a common method for investigating popular research directions and fields. **Figure 7A** shows the co-occurrence network visualization map of keywords in cluster view generated by CiteSpace. Through combining the synonyms and analogous keywords and changing g-index from k = 25 to k = 12, a clearer view of the keyword distribution was obtained.

As a result, a total of 319 keywords and 598 links extracted from all 1690 publications were visualized and analyzed. The nodes represent keywords, whose sizes are proportional to the frequency of keywords’ occurrence, and the time span of keywords ranges from January 1st, 2003, to July 31st, 2023. Each node consists of multiple concentric circles, whose color represents the publication time of the article containing the corresponding keywords, and the width of the circles represents the number of articles published within specific time frame. The link between nodes indicates that two keywords appear in the literature simultaneously, and the thickness of the link indicates the frequency of their occurrence, which is known as the degree of association. The top ten keywords (**Table 4**) are rheumatoid arthritis (1276 times), expression (545 times), fibroblast-like synoviocyte (322 times), microRNA (303 times), cell (226 times), systemic lupus erythematosus (197 times), inflammation (186 times), t cell (157 times), activation (151 times), nf kappa B (144 times). Evidently, research related to non-coding RNA in rheumatoid arthritis primarily involves the altered expression of disease-related genes in fibroblast-like synoviocytes and the activation of immune cells, specifically T cells. These events commonly lead to a series of inflammatory responses which closely associated with the NF-κB pathway.

**Table 4:** The top 20 frequency keywords regarding ncRNA and RA research from 2003-01-01 to 2023-07-31.

Rank	Keywords	Frequency	Rank	Keywords	Frequency
1	rheumatoid arthritis	1276	11	long noncoding rna	143
2	expression	684	12	proliferation	136
3	fibroblast-like synoviocyte	322	13	cancer	131
4	micro rna	303	14	disease	128
5	cell	226	15	tnf alpha	125
6	systemic lupus erythematosus	197	16	gene	116
7	inflammation	186	17	pathogenesis	109
8	t cell	157	18	altered expression	107
9	activation	151	19	autoimmune disease	103
10	nf kappa b	144	20	apoptosis	99

**Figure 7B** presented the visualization map of the keyword evolution in timeline view. The research on topics such as long noncoding RNA, synoviocyte, invasion, miR-146a, and circular RNA has received considerable attention. The keywords with the strongest citation burst are another crucial sign of research frontiers, hotspots, and emerging trends over time. The keywords such as “circular RNA” (2018-2023), “proliferation” (2014-2023), “migration” (2017-2023), and “oxidative stress” (2019-2023) burst continued in 2023, indicating that they are currently hot topics.

In the keywords analysis by CiteSpace, some ncRNAs (especially miRNAs and lncRNAs) had been investigated by many publications, revealing their crucial roles in the onset and diagnosis of RA. **Table 5** lists some of the mechanisms of these ncRNAs in the pathogenesis of RA. Clearly, RA-FLSs, with their aberrant proliferation, migration, invasion functions and the ability to secrete a substantial amount of inflammatory factors, play a significant role in the pathogenesis of RA. Additionally, it is worth noting that the keyword analysis did not comprises circRNAs mentioned in several studies. The primary reason is that research on circRNAs in RA is still in nascent stage, requiring additional time to accumulate more relevant research findings. Therefore, we can anticipate more studies in the future elucidating the specific roles of circRNAs in RA.

**Table 5:** The important ncRNAs identified through citespace’s keyword analysis that are associated with the pathogenesis of RA.

NcRNAs	Research subject	Expression	Targets/Regulators	Function	Ref.
miRNAs					
miR-146a	PBMCs, FLS	Up	–	promoted secretion of the inflammatory cytokines	(21, 22)
miR-155	PBMCs	Up	SOCS1	upregulation of TNF-α and IL-1β	(23)
	FLS		FOXO3a	promoted proliferation of RA-FLSs and secretion of the inflammatory cytokines	(24)
miR-21	FLS	Up	NF-κB	promoted the proliferation of RA-FLSs	(25)
miR-182	Bone marrow-derived macrophages (BMMs)	Up	Foxo3 and Maml1	miR-182 is identified as a crucial positive regulator of osteoclastogenesis	(26)
			PKR/IFN-β		(27)
miR-126	FLS	Up	PIK3R2	promotes proliferation and enhances resistance to apoptosis in RA-FLSs	(28)
			IL-23R	promoted the production of TNF-α and IFN-γ	(29)
miR-34a	Extracellular vesicles (Evs)	Up	cyclin I/ATM/ATR/p53	reduces the abnormal growth and inflammation of RA-FLSs	(30)
miR-140-5p	FLS	Down	STAT3	promotes proliferation and inflammatory cytokine expression while suppressing apoptosis in RA-FLSs	(31)
			TLR4	promotes the proliferation and secretion of IL-6, and IL-8 in RASFs	(32)
miR-27a	FLS	Down	FSTL1/TLR4/NF-κB	promotes migration and invasion of RA-FLSs	(33)
miR-223	The system of osteoclastogenesis from human PBMC cocultured with RA-FLS	Up	NFI-A, IL-17RD	overexpression of miR-223 suppresses osteoclastogenesis seen in RA	(34)
miR-451	neutrophils	Down	CPNE3, Rab5a	overexpression of miR-451 suppresses neutrophil chemotaxis via p38 MAPK	(35)
lncRNAs					
lncRNA GAS5	FLS	Down	miR-222-3p/Sirt1	overexpression of GAS5 suppresses proliferation, migration, inflammation, and promotes apoptosis of RA-FLSs	(36)
			miR-128-3p/HDAC4		(37)
			IL-18	overexpression of GAS5 induces apoptosis of RA-FLSs	(38)
lncRNA NEAT1	CD4+ T	Up	STAT3	knockdown of NEAT1 positively inhibits Th17/CD4+ T cell differentiation	(39)
	FLS		miR-410-3p/YY1	promoted migration, invasion, and inflammatory cytokines secretion in RA-FLSs	(40)
lncRNA H19	FLS	Up	TAK1	promotes the expression of inflammatory cytokines by activating the NF-κB and JNK/p38 MAPK pathways	(41)
lncRNA HOTAIR	serum samples	Up	IL-6 and MMP-9	promotes secretion of IL-6 and MMP-9	(42)

## 4 Discussion

### 4.1 General information of ncRNA-related RA research

This study performed a systematic literature search of publications on ncRNA-related RA research from January 1, 2003, to July 31, 2023, in the SCI-Expanded database of WOSCC. After excluding publications that did not meet the inclusion criteria, this bibliometric study comprised 1697 English papers published in 504 journals from 1872 institutions in 61 countries. **Figures 2A, B** show that the number of publications was generally on the rise from 2003 to 2023, which suggests that the research has promising prospects and potential. Although there are no wide variations in the number of publications in the past three years, a new burst may appear in “circular RNA”, “oxidative stress”, “proliferation” and “inflammation” in just a few years.

China has the highest number of publications, citations, and H-Index among all 61 countries, with all the top ten institutions located in China, indicating that China has significant influence in this field. However, the United States, with approximately one-third of the publication count of China, has achieved a similar H-Index, the highest level of collaboration, and the highest average number of citations per paper compared to China, suggesting that the United States continues to dominate in ncRNA-related RA research. This may be attributed to China’s academic evaluation system placing excessive emphasis on the quantity of SCI papers while overlooking quality and innovation, which has resulted in repetitive research lacking innovation. Additionally, there is relatively less international collaboration among Chinese institutions, which also limits China’s influence in the international academic community. Therefore, Chinese institutions and scholars need to focus more on conducting high-quality and innovative research on issues of significant scientific and societal value and expand international cooperation to increase their impact. In addition, with China’s economic development and increasing financial support for medical research, it is foreseeable that China’s academic productivity will continue to rise, and its influence on global academic research will become increasingly significant.

Based on the results of the top ten most productive journals, Frontiers In Immunology and International Journal Of Molecular Sciences can provide a wealth of high-quality literature resources for scholars who seek the latest frontier knowledge on ncRNA-related RA research. The top ten most influential co-cited journals include Arthritis & Rheumatology, Annals Of The Rheumatic Diseases, Nature, Cell, and Proceedings Of The National Academy Of Sciences Of The United States Of America, which cover a wide range of disciplines such as rheumatology, immunology, cell biology, and biochemistry. This indicates that ncRNA is widely recognized and studied in both basic and clinical research, which holds great research prospects significant prospects for RA diagnosis and treatment.

As for the authors, Lu, Qian-Jin is the most productive author and has the highest H-index, who provided an overview of the relationship between the dysregulation of miRNAs and the pathogenesis of autoimmune diseases, as well as the potential applications of miRNAs as biomarkers and therapeutic targets in autoimmune diseases (43–46), which provides insights into miRNA-related RA research.

### 4.2 Hotspots and emerging frontiers in ncRNA-related RA research

The visualization analysis of keywords (**Figure 6C**) in miRNA-related RA research by CiteSpace reveals that the latest hot research keywords primarily include “circular RNA”, “oxidative stress”, “proliferation”, and “migration”, and research in these areas has emerged as new hotspots in RA.

Specifically, researchers delved into the biological significance of ncRNAs in RA. In this field, circRNAs have emerged as a new focal point in gene expression regulation owing to its unique circular structure and enhanced stability. In RA, ncRNAs play a crucial role in regulating key cellular processes, encompassing immune response, inflammation, oxidative stress, and tissue damage (**Table 5**) (47). This emphasizes the potential of ncRNAs as more effective targets for the clinical treatment of RA. The proliferation, migration, and oxidative stress of pathological cells have garnered significant attention as critical steps in disease progression. Additionally, ncRNAs demonstrate specific expression patterns and high stability in disease conditions, making them precisely detectable in certain bodily fluids (such as blood, urine, etc.) (48). Therefore, ncRNA holds significant clinical value in the specific diagnosis of RA.

Overall, these studies lay the foundation for a deeper understanding of the pathogenesis of RA, the development of new treatment strategies, and the application of precision medicine. The continuous and in-depth research in these areas is expected to provide a more comprehensive understanding and effective intervention methods for future rheumatology research and clinical practice.

#### 4.2.1 The role and diagnostic value of circRNA in RA

RA is a severe chronic autoimmune disease that leads to joint inflammation and damage, as well as systemic impairment, which significantly impact patients’ health and quality of life. The early diagnosis and treatment of RA play a crucial role in delaying its progression, helping to prevent irreversible joint damage and disability (49).

The current diagnosis of RA is primarily based on the classification criteria of the American College of Rheumatology (ACR)/European League Against Rheumatism (EULAR) and certain serological markers, including rheumatoid factor (RF) and Anti-cyclic citrullinated peptide antibody (ACPA) (50). However, the limited sensitivity and specificity of these diagnostic markers often result in misdiagnosis and poor prognosis for RA patients (51). CircRNAs are ncRNAs that have been investigated extensively in RA following miRNAs and LncRNAs. Compared to traditional linear RNAs, circRNAs form a covalently closed loop structure which lacks 3’ PolyA tails and 5’ caps, making them effectively resistant to degradation by the ribonuclease R (RNase R) (52). Furthermore, circRNAs exhibit characteristics such as tissue specificity, conservation, and ubiquitous expression (53–56). Due to these characteristics, circRNAs hold greater potential as a diagnostic biomarker for diseases (48, 57–59). Several studies (**Table 6**) had explored the circRNA expression profiles in peripheral blood mononuclear cells of RA patients by microarray assay and receiver operating characteristic (ROC) analysis, and identified circRNAs such as hsa_circ_0005008, hsa_circ_0140271, and hsa_circ_101328 exhibit specificity and sensitivity in RA, which indicates that circRNAs hold significant promise as a diagnostic biomarker in RA with broad application prospects (9, 67, 68).

**Table 6:** AUC for circRNAs in PBMCs in discriminating RA patients from healthy controls.

CircRNA	Expression	AUC	Sensitivity	Specificity	References
CircRNA_104871	Up	0.833	0.833	0.68	(60)
CircRNA_003524	Up	0.683	0.833	0.6	
CircRNA_101873	Up	0.676	0.667	0.68	
CircRNA_103047	Up	0.671	0.667	0.56	
Hsa_circ_0000396	Down	0.809	–	–	(61)
Hsa_circ_0130438	Down	0.774	–	–	
Hsa_circ_0044235	Down	0.779	–	–	(62)
Hsa_circ_0035197	Up	0.742	0.712	0.686	(63)
Hsa_circ_0000367	Up	0.713	0.542	0.829	
Hsa_circ_0001947	Up	0.709	0.509	0.886	
Hsa_circ_0002715	Up	0.686	0.576	0.771	
Hsa_circ_0000175	Down	0.835	0.862	0.733	(64)
Hsa_circ_0008410	Up	0.806	0.552	0.956	
Hsa_circ_0003353	Up	0.897	0.832	0.689	(65)
Hsa_circ_0005732	Down	0.803	0.798	0.676	
Hsa_circ_0072428	Down	0.721	0.652	0.616	
CircPTPN22	Down	0.934	–	–	(66)
Hsa_circ_101328	Down	0.957	0.952	0.95	(9)

Furthermore, circRNAs offer new perspectives and insights in research related to the pathogenesis and treatment of RA (69). Current studies are primarily focused on the competitive endogenous RNA (ceRNA) mechanism of circRNAs. CircRNAs can function by sponging miRNAs to reduce the number of miRNAs available to target mRNA, thus contributing to mRNA stability or protein expression, which in turn influences the onset and development of RA (70). CircRNAs act as ceRNAs in various cell types associated with RA, including macrophages, helper T cells, chondrocytes, fibroblast-like synoviocyte (FLS), and others, and fibroblast-like synoviocytes are the primary focus of research in this context (**Table 7**). For example, CircRNA_09505 can promote AKT1 expression by acting as a miR-6089 sponge through the IκBα/NF-κB signaling pathway in macrophages, resulting in increased the production of TNF-α, IL-6, and IL-12, which cause joint inflammation and joint damage (77); CircNUP214 functioned as a miR-125a-3p sponge in RA, which promotes the expression of IL-17A and IL-23R in PBMCs and increases the proportion of Th17 cells, resulting in immune dysregulation and inflammatory responses (92); CircFADS2 promotes the expression of type II collagen, MMP-13, COX-2, and IL-6 via miR-498/mTOR axis, which leads to extracellular matrix (ECM) degradation, inflammation, and cell apoptosis in chondrocytes (72); Circ-PTTG1IP can promote TLR4 expression by acting as a miR-671-5p sponge, promoting the proliferation, migration, invasion and inflammatory response of FLSs in RA (89).

**Table 7:** CircRNAs related to the Pathogenesis of RA.

CircRNA	Expression	Distribution	Classification	Model	Targets	Research subject	References
Hsa_circ_0001859	Up	–	–	Hsa_circ_0001859/miR-204-5p、miR-211/ATF2	ATF2	SW982	(71)
CircFADS2	Up	–	–	CircFADS2/miR-498/mTOR	mTOR	Chondrocytes	(72)
CiRS-7	Up	–	–	CiRS-7/miR-7/mTOR	mTOR	PBMCs	(73)
Hsa_circ_0088036	Up	cytoplasm	exon	Hsa_circ_0088036/miR-140−3p/SIRT1	SIRT1	FLS	(74)
				Hsa_circ_0088036/miR-326/FZD4	FZD4		(75)
				Hsa_circ_0088036/miR-1263/REL/NF-κB	REL		(76)
CircRNA_09505	Up	–	–	CircRNA_09505/miR-6089/AKT1/NF-κB	AKT1	Synovial macrophages	(77)
CircHIPK3 (Hsa_circ_0000284)	Up	cytoplasm	–	CircHIPK3/miR-149-5p/FOXO1/VEGF	FOXO1	FLS	(78)
Circ-FAM120A (Hsa_circ_0003972)	Up	cytoplasm	–	Circ_0003972/miR-654-5p/FZD4	FZD4	FLS	(79)
				Circ-FAM120A/miR-671-5p/MDM4	MDM4		(80)
Circ_0025908	Up	–	–	Circ_0025908/miR-137/HIPK2	HIPK2	FLS	(81)
			exon	circ_0025908/miR-650/SCUBE2	SCUBE2	FLS	(82)
Circ_0088194	Up	cytoplasm	exon	Circ_0088194/miR-766-3p/MMP2	MMP2	FLS	(83)
Circ_AFF2 (Hsa_circ_0001947)	Up	cytoplasm	exon	Circ_AFF2/miR-375/TAB2	TAB2	FLS	(84)
				Circ-AFF2/miR-650/CNP	CNP		(85)
				CIRC_0001947/miR-671-5p/STAT3	STAT3		(86)
CircASH2L (Hsa_circ_0083964)	Up	–	exon	CircASH2L/miR-129-5p/HIPK2	HIPK2	FLS	(87)
CircMAPK9 (Hsa_circ_0001566)	Up	–	exon	CircMAPK9/miR-140-3p/PPM1A	PPM1A	FLS	(88)
Circ-PTTG1IP (Hsa_circ_0001200)	Up	cytoplasm	–	Circ-PTTG1IP/miR-671-5p/TLR4	TLR4	FLS	(89)
		–	exon	CircPTTG1IP/miR-431-5p/FSTL1	FSTL1	FLS	(90)
CircEDIL3 (Hsa_circ_0073244)	Up	cytoplasm	–	CircEDIL3/miR-485-3p/PIAS3/STAT3/VEGF	PIAS3	FLS and HDMEC	(91)
CircNUP214 (Hsa_circ_0089172)	Up	cytoplasm	exon	CircNUP214/miR-125a-3p/IL-23R	IL-23R	PBMCs and CD4^+^ T cell	(92)
Hsa-circ-0003353	Up	cytoplasm	exon	Hsa-circ-0003353/microRNA-31-5p/CDK1	CDK1	FLS	(93)
Circ_0088194	Up	–	–	Circ_0088194/miR-30a-3p/ADAM10	ADAM10	FLS	(94)
Circ_0004712	Up	–	–	Circ_0004712/miR-633/TRAF6	TRAF6	FLS	(95)
CircPTN	Up	cytoplasm	exon	CircPTN/miR-145-5p/FZD4	FZD4	FLS	(96)
Circ_0083964	Up	–	–	Circ_0083964/miR-204-5p/YY1	YY1	FLS	(97)
m6A-hsa_circ_0007259	Up	–	–	m6A-hsa_circ_0007259/hsa_miR-21-5p/STAT3	STAT3	Synovial tissues	(98)
CircCDKN2B-AS_006	Up	cytoplasm	–	CircCDKN2B-AS_006/miR-1258/RUNX1	RUNX1	FLS	(99)
Circ_0000479	Up	cytoplasm	–	Circ_0000479/miR-766/FKBP5	FKBP5	FLS	(100)
Circ_0002984	Up	cytoplasm	exon	Circ_0002984/miR-543/PCSK6	PCSK6	FLS	(101)
Circ_0000175	Up	cytoplasm	exon	Circ_0000175/miR-31-5p/GSDME	GSDME	FLS	(102)
Circ-Sirt1	Down	–	–	Circ-Sirt1/miR-132/SIRT1	SIRT1	FLS	(103)
Hsa_circ_0044235	Down	–	–	Hsa_circ_0044235/MiR-135b-5p/SIRT1	SIRT1	FLS	(104)
CircFBXW7	Down	cytoplasm	–	CircFBXW7/miR-216a-3p/HDAC4	HDAC4	FLS	(105)
Circ_0008360	Down	cytoplasm	–	Circ_0008360/miR-135b-5p/HDAC4	HDAC4	FLS	(106)
Circ_0130438(circKPNA5)	Down	–	exon	Circ_0130438/miR-130a-3p/KLF9	KLF9	FLS	(107)
Circ_0066715	Down	–	–	Circ_0066715/miR-486-5p/ETS1	ETS1	FLS and Macrophage	(108)
CircRNA_17725	Down	cytoplasm	–	CircRNA_17725/miR-4668-5p/FAM46C	FAM46C	Raw264.7	(109)
Circ_0000396	Down	–	exon	Circ_0000396/miR-574-5p/RSPO1	RSPO1	FLS	(110)

Based on the extensive research on circRNAs, researchers are further exploring the clinical applications of circRNAs as therapeutic targets. Compared to mRNA, miRNA, or lncRNA, circRNAs exhibit higher stability in the cytoplasm as therapeutic targets (111, 112). Additionally, circRNAs have lower immunogenicity of unmodified transcripts which will help to avoid activation of the innate immune responses that can decrease gene editing capabilities and disrupt protein production (113, 114). These two advantages can fully realize the potential of circRNAs in gene editing therapies and long-term protein expression regulation (115). Recent research has found that exosomes from circEDIL3-overexpressing synovial mesenchymal stem cells (SMSCs) can reduce VEGF expression by acting as a miR-485-3p sponge through the PIAS3/STAT3 signaling pathway in RA-FLS, and suppress inflammation-induced angiogenesis, which promotes pannus formation, ultimately ameliorated RA (91).

Consequently, circRNAs have started to exhibit the therapeutic potential *in vitro*. However, the application of circRNAs in clinical settings still requires further research, including how to construct more efficient and specific carriers for the delivery of circRNAs to specific cells or tissues. Additionally, it is important to clarify circRNAs’ pharmacokinetics, biodistribution in the body, and adverse reactions (116).

#### 4.2.2 Oxidative stress is a pivotal factor in rheumatoid arthritis progression, modulated by ncRNAs

Oxidative stress is characterized by the disruption of the intracellular redox balance, resulting in an excessive production of reactive oxygen species (ROS), leading to protein and DNA degradation, as well as lipid peroxidation (117). It plays a crucial role in accelerating the progression of RA and exacerbating clinical symptoms, which includes triggering immune cell activation, promoting synovial inflammation, inducing cell apoptosis and necrosis, increasing joint pain, and initiating joint damage (118–121). Research has revealed that the inflammatory synovial cavity in RA patients is a severely hypoxic environment, which serves as the primary site for oxidative stress and is the fundamental condition for the accumulation of ROS and mitochondrial damage (122). FLS play a pivotal role in the pathogenesis of RA (123). Under hypoxic conditions, FLS exhibit an increased frequency of mitochondrial DNA (mtDNA) mutations, leading to mitochondrial dysfunction, which results in the downregulation of mitochondrial respiration and a decrease in ATP production, along with an upregulation of glycolysis and the accumulation of ROS (124).

Oxidative stress directly activates redox-sensitive transcription factors such as NF-κB, Hypoxia-inducible factor-1α (HIF-1α), Activator Protein-1 (AP-1), and Nuclear factor erythroid2-related factor 2 (Nrf2), which exacerbate the inflammatory responses, oxidative stress, proliferation, invasion, and migration of FLS, induce the recruitment and activation of macrophages and neutrophils that contributing to oxidative stress and inflammatory responses, as well as directly or indirectly contribute to cartilage and bone damage (125–130).

ROS can induce the tyrosine phosphorylation of IκBα by activating Syk kinase, which leads to the dissociation, phosphorylation, and nuclear translocation of NF-κB p65 subunit, thereby stimulating the NF-κB pathway (131, 132). In RA, NF-κB is involved in the induction of various pro-inflammatory cytokines and chemokines, including TNF-α, IL-1β, IL-6, GM-CSF, and others, within monocytes, macrophages, and synovial cells (133). Consequently, these cytokines recruit additional immune cells from the immune system and activate the NF-κB factor, resulting in the initiation of an inflammatory loop (134–136). Furthermore, NF-κB also participates in the regulation of Cyclin D1, Matrix Metalloproteinases (MMPs), and Vascular Endothelial Growth Factor (VEGF), which contribute to the proliferation, migration, and invasion of FLS, as well as cartilage tissue damage (137–139).

HIF-1α is highly regulated by oxygen levels, and in the hypoxic environment of the synovium, HIF-1α accumulates and translocates to the nucleus, where it forms a heterodimer with HIF-1β, known as HIF-1 (140, 141). HIF-1 binds to the HRE promoter thus mediating VEGF expression, thereby leading to angiogenesis (142). Moreover, the upregulation of HIF-1α and VEGF leads to an increase in the expression of stromal cell–derived factor 1α (SDF1α)/CXCL12 mRNA in RA-FLS, which facilitates the recruitment of leukocytes to RA synovial tissue and triggers the release of matrix metalloproteinase 3 (MMP-3) by chondrocytes (143). Furthermore, through the overexpression of HIF-1α in FLS, the upregulation of MMP-1, MMP-3, and IL-8 expression was induced, indicating that HIF-1 can recruit neutrophils, exacerbate inflammation, and induce bone and cartilage damage (144). Studies had found that HIF-1α also plays a significant role in bone cells. Under hypoxic conditions, HIF-1α can upregulate receptor activator of nuclear factor‐κB ligand (RANKL) expression through the JAK2/STAT3 pathway, thereby inducing osteocyte‐mediated osteoclastogenesis (145). HIF-1α can also reduce osteoclast iron death by inhibiting osteoclast iron-starvation response and RANKL-induced ferritinophagy (146). Additionally, HIF-1α can increase the expression and release of angiopoietin-like 4 (ANGPTL4) in various cell types within the rheumatoid synovium, thereby promoting osteoclast-mediated bone resorption (147, 148).

AP-1 is typically a homodimer/heterodimer composed of c-Jun and c-Fos proteins. ROS can activate AP-1 in RA-FLS, leading to the upregulation of IL-1β, MMP-1, MMP-3, and MMP-9 expression (149, 150). Additionally, AP-1 promotes cPLA2 expression by binding to the cPLA2 promoter region, and subsequently increasing the production of eicosanoids like prostaglandin E_2_ (PGE_2_), which contributes to inflammation and exacerbates pain (151).

Nrf2 is a crucial factor in the RA antioxidant system, regulating the expression of multiple antioxidant genes (152–154). It is also an important target in current research on ncRNA regulation of oxidative stress in RA. In the cytoplasm, Kelch-like ECH-associated protein-1 (keap1) binds to Nrf2 and promotes its degradation, while ROS can directly activate Nrf2, leading to the dissociation of Nrf2 from Keap1 and its translocation into the cell nucleus, where it interacts with antioxidant response elements (ARE), such as heme oxygenase-1 (HO-1) and superoxide dismutase (SOD), to exert its antioxidant effects (155–157). Currently, multiple studies have revealed the involvement of ncRNAs in regulating Nrf2 expression in RA. For instance, overexpression of miR-30a-3p in RA-FLS of rat can downregulate the expression of Keap1 and cullin 3 (cul3), promoting the expression of Nrf2 and its downstream targets, HO-1 and NAD(P)H: Quinone Oxidoreductase 1 (NQO1), thus exerting antioxidant effects (158). Additionally, overexpression of LINC00638 in RA-FLS activates the Nrf2/HO-1 pathway, leading to the upregulation of SOD2 expression and a reduction in levels of ROS and reactive nitrogen species (RNS) (159).

NcRNAs have the capability to regulate the expression of multiple target genes. Currently, extensive and comprehensive studies have been conducted on ncRNAs in the context of synovial inflammation, vascular angiogenesis, and the destruction of bone and cartilage joints in RA. However, much remains to be discovered about oxidative stress in RA. Oxidative stress can expedite the progression of RA by intensifying the inflammatory loop and causing irreversible joint damage. Hence, the exploration of ncRNAs’ role in regulating oxidative stress in RA shows promise in offering new potential therapeutic approaches to manage the advancement of the disease.

#### 4.2.3 NcRNAs influence the development of RA by regulating the proliferation, migration, and invasion of FLS

FLSs are the predominant cells in synovial tissue, and in RA, they exhibit a high proliferative capacity and an aggressive pathogenic phenotype, invading joint cartilage and bone while secreting MMPs, resulting in joint damage (123, 160, 161). Consequently, it is important to target RA-FLS to suppress their proliferation, migration, and invasive capabilities to counteract joint damage.

Currently, several studies have revealed that ncRNAs play a role in regulating proteins associated with proliferation, migration, and invasion in RA-FLS. MiRNAs can bind to complementary base sequences on target mRNA molecules, resulting in decreased stability, degradation, or inhibition of the translation process of the target mRNA, ultimately reducing the expression level of the target protein (162). For example, miR-4423-3p inhibits the proliferation, migration and invasion of RA-FLS by downregulating the expression level of MMP13 (163); MiR-449a downregulates the expression of High Mobility Group Box 1 (HMGB1) to inhibit the proliferation, migration, and invasion of RA-FLS (164); MicroRNA-133 downregulates mesenchymal-epithelial transition factor (MET), Epidermal Growth Factor Receptor (EGFR), and Fascin Actin-Bundling Protein 1 (FSCN1) to suppress the proliferation, migration, and invasion of RA-FLS (165).

LncRNAs and circRNAs primarily act as sponges for miRNAs, indirectly regulating the expression of target gene mRNAs, thereby promoting the progression of RA (166, 167). Circ_0088194 regulates proliferation, migration, and invasion in RA-FLS through the miR-30a-3p/ADAM10 axis (94). Long non-coding RNA NR-133666 promotes proliferation and migration in RA-FLS through the miR-133c/MAPK1 axis (168). In addition, LncRNAs can also form complexes with proteins, collaboratively participating in the regulation of specific gene expression (166). The elevated expression of LncRNA HAFML in RA-FLS promotes the formation of the HAFML-HuR complex, facilitating the binding of HuR (Human antigen R) to APPL2 mRNA and enhancing its translation, which promotes the migration and invasion of RA-FLS (169). The decreased expression of LncRNA LERFS in RA-FLS leads to a reduction in the complex formation between LERFS and heterogeneous nuclear ribonucleoprotein Q (hnRNP Q), which reduces the binding of the complex to RhoA, Rac1, and Cdc42 mRNA, subsequently promoting the translation process and leading to the proliferation, migration, and invasion of RA-FLS (170).

Evidently, non-coding RNAs (ncRNAs) can effectively modulate the proliferation, migration, and invasion capacities of RA-FLSs through various pathways and targets, thus contributing to the reduction of irreversible damage to joint cartilage and bone (167). Moreover, drugs specifically tailored to target the biological functions of RA-FLS can be locally administered, enabling higher drug concentrations and prolonged therapeutic durations, which enhances treatment efficacy while reducing systemic drug distribution, thereby minimizing adverse effects on other tissues and organs (116, 171). This comprehensive therapeutic strategy represents a promising avenue for RA management.

### 4.3 Summary and prospects of ncRNA-Related Research in RA

The second part of the discussion primarily emphasizes the role of ncRNAs (particularly circRNAs) in oxidative stress in RA and its impact on the biological functions of RA-FLSs, including proliferation, migration, and invasion. Here, I will summarize the ncRNAs (miRNA and lncRNA) in the pathogenesis of RA that haven’t been extensively detailed in this text.

#### 4.3.1 miRNAs in the pathogenesis of RA

MiRNAs are small, single-stranded transcripts of about 18-24 nucleotides in length. They do not directly encode proteins, instead, they regulate gene expression at both the transcriptional and translational levels (172, 173).

Several studies have indicated abnormal expression of miRNAs in blood or tissue samples from RA patients, suggesting their potential role in the pathogenesis of RA. As listed in **Table 5**, miR-146a and miR-155 had been consistently identified through multiple studies as being overexpressed in PBMCs (21, 23, 174–176). However, isolating PBMCs and fresh plasma is not a common practice in routine clinical procedures, and there is a possibility of significant alteration in miRNA biomarker level caused by minor variations in cell counts or hemolysis during the isolation of cell-free plasma or blood-derived immune cells (such as PBMCs) (177, 178). Based on these two points, further research has revealed a strong correlation between whole blood samples and PBMC expression levels of miR-155 and miR-146 in healthy controls and RA patients (179). This discovery offers a new perspective on the application of miRNAs as clinical biomarkers in RA, facilitating the use of miRNA-based diagnostics in routine clinical practice by using easily accessible samples, such as whole blood. Moreover, recent evidence has demonstrated a promising platform for detecting certain cancers through profiling miRNAs derived from whole blood (180, 181). This significantly broadens the scope for the application of miRNAs as clinical biomarkers.

Additionally, miRNAs are also aberrantly expressed in RA-FLSs and regulate the inflammatory responses, proliferation, migration, and invasion of RA-FLSs. For example, miR-21 promotes the proliferation of RA-FLS by mediating NF-κB nuclear translocation (25); overexpression of miR-27a inhibits migration and invasion of RA-FLS and simultaneously decreases the expression of MMP2, MMP9, and MMP13 via the FSTL1/TLR4/NF-κB axis (33); miR-34a can be transported to RA-FLS via extracellular vesicles (EVs), which reduced inflammation, proliferation, and resistance to apoptosis of RA-FLS by inhibiting the cyclin I/ATM/ATR/p53 signaling pathway (30). MiRNAs can also regulate immune cells and the differentiation of bone cells to modulate the progression of RA. For instance, miR-146a enhances a pro-inflammatory phenotype in Tregs by upregulating the expression of STAT1 (182); miR-182 had been identified as a critical positive regulator of osteoclastogenesis, which is associated with the Foxo3, Maml1 and PKR/IFN-β signaling pathways (26, 27).

#### 4.3.2 LncRNAs in the pathogenesis of RA

LncRNAs are transcripts with a length larger than 200 nucleotides that regulate gene expression through various mechanisms, including epigenetic regulation, transcriptional control, and post-transcriptional regulation. The regulatory mechanisms of lncRNAs mainly encompass (183, 184): (1) LncRNAs can act as sponges for miRNAs, indirectly regulating the expression of target gene mRNAs; (2) LncRNAs can interact with proteins, influencing the binding of proteins to chromatin DNA, altering chromatin structure, and affecting the activity of transcription factors, thus regulating gene transcription; (3) LncRNAs recruit chromatin-modifying enzymes (such as methyltransferases and deacetylases) to specific gene regions, altering histone modification patterns, and modulating chromatin status and gene expression; (4) LncRNAs can form complexes with transcription factors or other RNAs, serving as part of ribonucleoprotein complexes, recruiting other proteins or RNAs, thereby influencing the formation and function of transcription complexes; (5) LncRNAs can regulate mRNA processing, splicing, stability, and translation in the post-transcriptional phase, affecting the final products of gene expression. Currently, the dysregulation of lncRNAs is also observed in the PBMCs, FLS, and immune cells of RA patients, suggesting that lncRNAs might influence the progression of RA by impacting the function of these cells.

**Table 5** shows the important LncRNAs associated with the pathogenesis of RA. LncRNA GAS5 can be upregulated by Tanshinone IIA, thereby enhancing the expression of caspase-3/caspase-9 and suppressing the PI3K/AKT signaling pathway, resulting in the apoptosis of RA-FLS (185). This demonstrates the functional capacity of LncRNAs as potential therapeutic targets and their effectiveness as biomarkers to evaluate treatment responses in RA patients. Shui X (39) exploring the dysregulated lncRNAs in PBMCs and differentiated Th17 cells of RA patients and observed an upregulation of LncRNA NEAT1. Additionally, knockdown of lncRNA NEAT1 inhibits Th17/CD4+ T cell differentiation through reducing the STAT3 protein level (39). LncRNA H19 expression was found to be higher in PBMCs of RA patients compared to healthy volunteers (186). Overexpression of H19 in PMA-induced THP-1 cells (a common model used to study macrophage activity modulation) upregulates KDM6A and various M1 macrophage-associated factors (particularly CCL8, CXCL9, CXCL10, and CXCL11) which promote M1 macrophage activity and macrophage migration (186). LncRNA H19 can also aggravate TNF-α-induced inflammatory injury via TAK1 pathway in MH7A cells (41). LncRNA Hotair exhibited notably high expression levels in the PBMCs and serum exosomes of RA patients, promoting the migration of activated macrophages (187). Meanwhile, markedly lower levels of LncRNA Hotair were detected in differentiated osteoclasts and RA-FLS, and upregulating LncRNA Hotair can decrease the activation of MMP-2 and MMP-13 in both differentiated osteoclasts and RA-FLS (187).

#### 4.3.3 Summary and prospects

In general, whether it’s miRNAs, lncRNAs, or circRNAs, their roles in the pathogenesis of RA are quite similar and can be summarized in the following three points: (1) ncRNAs regulates the inflammatory response of immune cells in the synovium (188); (2) the expression of ncRNAs influences the biological behavior of RA-FLSs, including affecting inflammation, proliferation, migration, and invasion functions; (3) ncRNAs participate in bone metabolism in RA, promoting the secretion of MMPs by RA-FLSs to disrupt the bone matrix and regulating the activation of osteoclasts. During the process in which non-coding RNA exerts its influence, miRNA, being relatively short, exhibits high specificity when binding to target genes. However, this characteristic also limits its diversity in molecular interactions. On the other hand, lncRNA, usually longer and forming stable secondary or tertiary structures, remains relatively stable in cells, resisting degradation by RNase R. Its more complex structure and diverse functional domains allow it to interact with multiple molecules. CircRNAs, share similar functions with lncRNAs, have a circular structure that provides an advantage in resisting degradation compared to linear RNA. While circRNAs and lncRNAs have become hot topics in research due to their structural and functional advantages, it is undeniable that the regulatory roles played by circRNA, lncRNA, and miRNA at the cellular and molecular levels should not be overlooked. Further clarification of the regulatory network constituted by circRNA, lncRNA, and miRNA will be a major research direction in the future (10).

In addition to understanding the role of ncRNAs in the pathogenesis of RA, exploring their application in the diagnosis and treatment of RA is equally crucial. The future application of ncRNAs in RA might develop in several aspects: (1) ncRNA as a disease biomarker for RA diagnosis; (2) ncRNA as a predictor of complications in RA patients, such as cardiovascular complications (189); (3) ncRNA as a biomarker for assessing the treatment response of RA patients (190); (4) the use of engineered exosomes containing ncRNA for RA treatment (13). Exploration in these areas is essential for advancing our comprehension of RA pathogenesis and enhancing diagnostic and treatment approaches.

## 5 Limitations

This study provides a bibliometric and visual analysis of the current research status and trends related to ncRNAs in RA. However, there are certain limitations to this research that need to be acknowledged. Firstly, the study exclusively gathered English articles and reviews from the WoSCC-SCI-EXPANDED database, with a literature collection cut-off date of July 31, 2023, potentially excluding some relevant publications. Secondly, software algorithms like Citespace and VOSviewer have limitations in that they cannot analyze the full text of publications, leading to the omission of certain details and some margin of error in the results. Thirdly, the total number of citations usually rises over time. Thus, early publications have a higher likelihood of being referenced compared to newly released ones, which enhancing the significance of information gathered from early publications in **Tables 2** and **8**. Fourthly, this study did not conduct a quality assessment of the included publications, treating all of them as having equal validity. It is hoped that future research can address these limitations by collecting literature data from additional databases and employing quality assessment methods to provide a more comprehensive and accurate understanding of non-coding RNA research in RA.

**Table 8a:** The top 10 most productive journals regarding ncRNA and RA research from 2003-01-01 to 2023-07-31.

Rank	Journal	Countries	Count	IF(2023)	JCR(2023)	Total citations	Average citation per paper	H-index
1	Frontiers In Immunology	Switzerland	75	7.3	Q1	2243	29.9067	26
2	International Journal Of Molecular Sciences	Switzerland	43	5.6	Q1	1041	24.2093	19
3	Plos One	United States	33	3.7	Q2	1209	36.6364	21
4	Autoimmunity	United Kingdom	30	3.5	Q3	550	18.3333	12
5	International Immunopharmacology	Netherlands	30	5.6	Q1	404	13.4667	15
6	Arthritis Research & Therapy	United Kingdom	28	4.9	Q2	2635	94.1071	24
7	Molecular Medicine Reports	Greece	26	3.4	Q3	582	22.3846	14
8	Scientific Reports	United Kingdom	24	4.6	Q2	841	35.0417	15
9	Clinical Rheumatology	Germany	23	3.4	Q3	410	17.8261	11
10	Journal Of Autoimmunity	United States	22	12.8	Q1	1624	73.8182	17

**Table 8b:** The top 10 co-cited journals regarding ncRNA and RA research from 2003-01-01 to 2023-07-31.

Rank	Co-cited Journal	Countries	Citations	IF(2023)	JCR(2023)
1	Arthritis & Rheumatology	United States	4696	13.3	Q1
2	Journal Of Immunology	United States	2457	4.4	Q2
3	Arthritis Research & Therapy	United Kingdom	2447	4.9	Q2
4	Annals Of The Rheumatic Diseases	United Kingdom	2432	27.4	Q1
5	Proceedings Of The National Academy Of Sciences Of The United States Of America	United States	2361	11.1	Q1
6	Plos One	United States	2263	3.7	Q2
7	Nature	United Kingdom	2104	64.8	Q1
8	Cell	United States	1870	64.5	Q1
9	Frontiers in Immunology	United States	1344	7.3	Q1
10	Journal Of Autoimmunity	United States	1320	12.8	Q1

## 6 Conclusion

Over the past two decades, as the role of non-coding RNAs in the pathogenesis of RA has been increasingly unveiled, their potential value in the diagnosis and treatment of RA has garnered growing attention. This study conducted a bibliometric analysis of literature related to ncRNAs in RA using various statistical analysis tools. It has revealed the countries, institutions, authors, and journals with the most significant impact in this field. Additionally, it has identified the hot trends in ncRNA research concerning the pathogenesis, diagnosis, and treatment of RA. Currently, circRNAs and the biological functions of RA-FLS have emerged as new research focal points. In the future, targeted drugs designed based on circRNA regulatory mechanisms may become potential therapeutic strategies for controlling the progression of RA by targeting RA-FLS. This trend underscores the importance of non-coding RNAs in the field of RA research and provides a promising direction for future therapeutic investigations.

## Data availability statement

Publicly available datasets were analyzed in this study. This data can be found here: https://www.webofscience.com/wos/woscc/summary/868a42b7-1929-4c31-8a06-3b183a5293a1-9ea62624/relevance/1.

## Author contributions

ZW: Data curation, Investigation, Software, Visualization, Writing – original draft, Writing – review & editing. HL: Methodology, Writing – review & editing. SL: Data curation, Investigation, Writing – review & editing. JY: Writing – review & editing, Funding acquisition, Project administration, Resources, Supervision.
